# Adult non-communicable disease mortality in Africa and Asia: evidence from INDEPTH Health and Demographic Surveillance System sites

**DOI:** 10.3402/gha.v7.25365

**Published:** 2014-10-29

**Authors:** P. Kim Streatfield, Wasif A. Khan, Abbas Bhuiya, Syed M.A. Hanifi, Nurul Alam, Cheik H. Bagagnan, Ali Sié, Pascal Zabré, Bruno Lankoandé, Clementine Rossier, Abdramane B. Soura, Bassirou Bonfoh, Siaka Kone, Eliezer K. Ngoran, Juerg Utzinger, Fisaha Haile, Yohannes A. Melaku, Berhe Weldearegawi, Pierre Gomez, Momodou Jasseh, Patrick Ansah, Cornelius Debpuur, Abraham Oduro, George Wak, Alexander Adjei, Margaret Gyapong, Doris Sarpong, Shashi Kant, Puneet Misra, Sanjay K. Rai, Sanjay Juvekar, Pallavi Lele, Evasius Bauni, George Mochamah, Carolyne Ndila, Thomas N. Williams, Kayla F. Laserson, Amek Nyaguara, Frank O. Odhiambo, Penelope Phillips-Howard, Alex Ezeh, Catherine Kyobutungi, Samuel Oti, Amelia Crampin, Moffat Nyirenda, Alison Price, Valérie Delaunay, Aldiouma Diallo, Laetitia Douillot, Cheikh Sokhna, F. Xavier Gómez-Olivé, Kathleen Kahn, Stephen M. Tollman, Kobus Herbst, Joël Mossong, Nguyen T.K. Chuc, Martin Bangha, Osman A. Sankoh, Peter Byass

**Affiliations:** 1 Matlab HDSS, Bangladesh; 2 International Centre for Diarrhoeal Disease Research, Bangladesh; 3 INDEPTH Network, Accra, Ghana; 4 Bandarban HDSS, Bangladesh; 5 Chakaria HDSS, Bangladesh; 6 Centre for Equity and Health Systems, International Centre for Diarrhoeal Disease Research, Bangladesh; 7 AMK HDSS, Bangladesh; 8 Centre for Population, Urbanisation and Climate Change, International Centre for Diarrhoeal Disease Research, Bangladesh; 9 Nouna HDSS, Burkina Faso; 10 Nouna Health Research Centre, Nouna, Burkina Faso; 11 Ouagadougou HDSS, Burkina Faso; 12 Institut Supérieur des Sciences de la Population, Université de Ouagadougou, Burkina Faso and Institut d’Études Démographique et du parcours de vie, Université de Genève, Geneva, Switzerland; 13 Taabo HDSS, Côte d'Ivoire; 14 Centre Suisse de Recherches Scientifiques en Côte d'Ivoire, Abidjan, Côte d'Ivoire; 15 Université Félix Houphoët-Boigny, Abidjan, Côte d'Ivoire; 16 Swiss Tropical and Public Health Institute, Basel, Switzerland; 17 Kilite-Awlaelo HDSS, Ethiopia; 18 Department of Public Health, College of Health Sciences, Mekelle University, Mekelle, Ethiopia; 19 Farafenni HDSS, The Gambia; 20 Medical Research Council, The Gambia Unit, Fajara, The Gambia; 21 Navrongo HDSS, Ghana; 22 Navrongo Health Research Centre, Navrongo, Ghana; 23 Dodowa HDSS, Ghana; 24 Dodowa Health Research Centre, Dodowa, Ghana; 25 Ballabgarh HDSS, India; 26 All India Institute of Medical Sciences, New Delhi, India; 27 Vadu HDSS, India; 28 Vadu Rural Health Program, KEM Hospital Research Centre, Pune, India; 29 Kilifi HDSS, Kenya; 30 KEMRI-Wellcome Trust Research Programme, Kilifi, Kenya; 31 Department of Medicine, Imperial College, St. Mary's Hospital, London, United Kingdom; 32 Kisumu HDSS, Kenya; 33 KEMRI/CDC Research and Public Health Collaboration and KEMRI Center for Global Health Research, Kisumu, Kenya; 34 Nairobi HDSS, Kenya; 35 African Population and Health Research Center, Nairobi, Kenya; 36 Karonga HDSS, Malawi; 37 Karonga Prevention Study, Chilumba, Malawi; 38 London School of Hygiene and Tropical Medicine, London, United Kingdom; 39 Niakhar HDSS, Senegal; 40 Institut de Recherche pour le Developpement (IRD), Dakar, Sénégal; 41 Agincourt HDSS, South Africa; 42 MRC/Wits Rural Public Health and Health Transitions Research Unit (Agincourt), School of Public Health, Faculty of Health Sciences, University of the Witwatersrand, Johannesburg, South Africa; 43 Umeå Centre for Global Health Research, Umeå University, Umeå, Sweden;; 44 Africa Centre HDSS, South Africa; 45 Africa Centre for Health and Population Studies, University of KwaZulu-Natal, Somkhele, KwaZulu-Natal, South Africa; 46 National Health Laboratory, Surveillance & Epidemiology of Infectious Diseases, Dudelange, Luxembourg; 47 FilaBavi HDSS, Vietnam; 48 Health System Research, Hanoi Medical University, Hanoi, Vietnam; 49 School of Public Health, Faculty of Health Sciences, University of the Witwatersrand, Johannesburg, South Africa; 50 Hanoi Medical University, Hanoi, Vietnam; 51 WHO Collaborating Centre for Verbal Autopsy, Umeå Centre for Global Health Research, Umeå University, Umeå, Sweden

**Keywords:** adults, non-communicable disease, Africa, Asia, mortality, INDEPTH Network, verbal autopsy, InterVA

## Abstract

**Background:**

Mortality from non-communicable diseases (NCDs) is a major global issue, as other categories of mortality have diminished and life expectancy has increased. The World Health Organization's Member States have called for a 25% reduction in premature NCD mortality by 2025, which can only be achieved by substantial reductions in risk factors and improvements in the management of chronic conditions. A high burden of NCD mortality among much older people, who have survived other hazards, is inevitable. The INDEPTH Network collects detailed individual data within defined Health and Demographic Surveillance sites. By registering deaths and carrying out verbal autopsies to determine cause of death across many such sites, using standardised methods, the Network seeks to generate population-based mortality statistics that are not otherwise available.

**Objective:**

To describe patterns of adult NCD mortality from INDEPTH Network sites across Africa and Asia, according to the WHO 2012 verbal autopsy (VA) cause categories, with separate consideration of premature (15–64 years) and older (65+ years) NCD mortality.

**Design:**

All adult deaths at INDEPTH sites are routinely registered and followed up with VA interviews. For this study, VA archives were transformed into the WHO 2012 VA standard format and processed using the InterVA-4 model to assign cause of death. Routine surveillance data also provide person-time denominators for mortality rates.

**Results:**

A total of 80,726 adult (over 15 years) deaths were documented over 7,423,497 person-years of observation. NCDs were attributed as the cause for 35.6% of these deaths. Slightly less than half of adult NCD deaths occurred in the 15–64 age group. Detailed results are presented by age and sex for leading causes of NCD mortality. Per-site rates of NCD mortality were significantly correlated with rates of HIV/AIDS-related mortality.

**Conclusions:**

These findings present important evidence on the distribution of NCD mortality across a wide range of African and Asian settings. This comes against a background of global concern about the burden of NCD mortality, especially among adults aged under 70, and provides an important baseline for future work.

Mortality due to non-communicable diseases (NCDs) of various kinds has become an increasing focus of attention in recent years (1), including at a special meeting of the UN General Assembly in September 2011 (2). This has happened both because of rapid increases in major NCD risk factors to which billions of people globally are exposed and also because many populations are ageing more successfully as a result of various reductions in early-life mortality and increases in mid-life survival. Consequently, more people globally are experiencing the consequences of NCD risk factors in middle and later life, according to the classic theories of epidemiological transition (3). In 2012, WHO Member States at the World Health Assembly made a resolution to ‘adopt a global target of a 25% reduction in premature mortality from non-communicable diseases by 2025’ (4), although this has been widely misquoted with the omission of the word ‘premature’. The biological reality is that human beings who survive into their 80s and 90s, thereby having avoided death from many other causes, are extremely likely to die of NCDs. The public health imperatives around NCDs therefore relate to preventing or delaying NCD incidence (including risk factor management) and effectively managing chronic conditions in the decades of mid-life.

There has been much written about epidemics of NCD morbidity and mortality globally, and particularly in low- and middle-income countries (5). While it is certainly true that there are many more NCD deaths now than there have been previously, it is critical to understand the medical, social, and demographic drivers of these changes, in addition to trends in specific risk factors, diseases, and health systems responses. This is important in terms of positioning the burden of NCDs appropriately in future development agendas (6).

The INDEPTH Network works with Health and Demographic Surveillance sites (HDSS) across Africa and Asia, which each follow circumscribed populations on a longitudinal basis. Core data collected include person-time at risk, together with deaths and, by means of verbal autopsy (VA), assessment of cause of death (7).

Our aim in this paper is to document deaths among adults (15 years upwards) on the basis of a dataset collected at 22 INDEPTH HDSSs covering Africa and Asia (8), looking particularly at those deaths attributable to NCDs. Although these 22 sites are not designed to be a representative sample, they enable comparisons to be made over widely differing situations.

## Methods

The overall INDEPTH dataset (9) from which these adult NCD mortality analyses are drawn is described in detail elsewhere (8). Across the 22 participating sites (10–31) , there was documentation on 80,726 deaths in 7,423,497 person-years of observation for people aged 15 and over. VA interviews were successfully completed on 72,330 (89.6%) of the deaths that occurred. A summary of the detailed methods used in common for this series of multisite papers is shown in Box 1. NCD mortality was defined as neoplasms, metabolic, cardiovascular, respiratory, abdominal, neurological and other NCDs, corresponding to chapters in WHO 2012 VA standard.

*Box 1.* Summary of methodology based on the detailed description in the introductory paper (8).

**Age–sex–time standardisation**

To avoid effects of differences and changes in age– sex structures of populations, mortality fractions and rates have been adjusted using the INDEPTH 2013 population standard (32). A weighting factor was calculated for each site, age group, sex, and year category in relation to the standard for the corresponding age group and sex, and incorporated into the overall dataset. This is referred to in this paper as age–sex-time standardisation in the contexts where it is used.

**Cause of death assignment**

The InterVA-4 (version 4.02) probabilistic model was used for all the cause of death assignments in the overall dataset (33). InterVA-4 is fully compliant with the WHO 2012 VA standard and generates causes of death categorised by ICD-10 groups (34). The data reported here were collected before the WHO 2012 VA standard was available, but were transformed into the WHO 2012 and InterVA-4 format to optimise cross-site standardisation in cause of death attribution. For a small proportion of deaths, VA interviews were not successfully completed; a few others contained inadequate information to arrive at a cause of death. InterVA-4 assigns causes of death (maximum 3) with associated likelihoods; thus, cases for which likely causes did not total to 100% were also assigned a residual indeterminate component. This served as a means of encapsulating uncertainty in cause of death at the individual level within the overall dataset, as well as accounting for 100% of every death.

**Overall dataset**

The overall public-domain dataset (9) thus contains between one and four records for each death, with the sum of likelihoods for each individual being unity. Each record includes a specific cause of death, its likelihood, and its age–sex–time weighting.

In this context, all of these data are secondary datasets derived from primary data collected separately by each participating site. In all cases, the primary data collection was covered by site-level ethical approvals relating to on-going health and demographic surveillance in those specific locations. No individual identity or household location data were included in the secondary data and no specific ethical approvals were required for these pooled analyses.

## Results

After age–sex–time standardisation, 81814.1 deaths were documented, of which 73288.7 were covered by VA interviews; the InterVA-4 model was unable to assign any cause of death for 3458.3 deaths (4.2%), and residual uncertainties (residual likelihoods in cases where likely cause(s) of death did not total to 100%) accounted for 5864.2 deaths (7.2%). As a consequence of sites returning data over a range of time periods, 4.6% of the person-time observed occurred before 2000, 30.0% from 2000 to 2005, and 69.4% from 2006 to 2012.

Non-NCD causes were attributed to 35478.5 deaths (43.4%), while 28487.7 deaths (34.8%) were attributed to 20 specific WHO 2012 cause of death categories, plus the residual ‘other and unspecified NCD’ category. Since the Purworejo, Indonesia, site achieved limited coverage of adult VAs, and did not report for the period 2006–2012, that site is excluded from the following analyses (1173.4 deaths in 185,306 person-years).

**Fig. 1:**
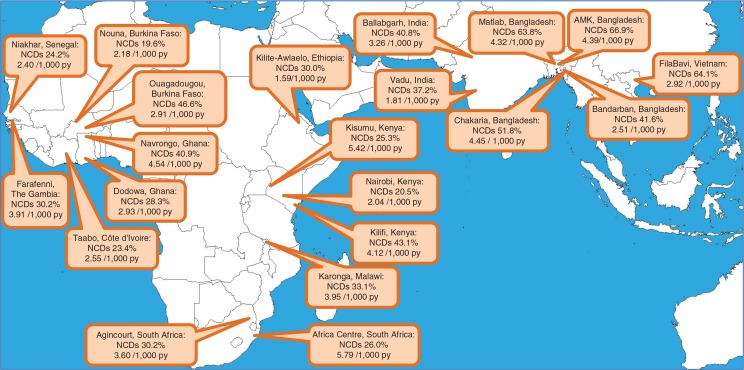
Map showing age–sex–time standardised proportions of mortality due to non-communicable diseases and age–sex–time standardised non-communicable disease mortality rates per 1,000 person-years, for 21 INDEPTH sites.

Figure 1 maps the 21 INDEPTH sites, showing the age–sex–time standardised NCD-specific fraction of adult mortality and the age–sex–time standardised NCD-specific mortality rate per 1,000 person-years for each site. This shows a relatively narrow range of variation across sites, while the sites with the highest proportions of NCD-attributable mortality are not necessarily those with the highest rates. Generally, higher proportions of NCD mortality, but not always higher rates, were found in Asia compared to Africa. The urban site in Ouagadougou, Burkina Faso, returned the highest proportion of NCD mortality in Africa (46.6%), while the Africa Centre, South Africa, site reported the highest rate of NCD mortality (5.79 per 1,000 person-years). The highest proportion and rate of NCD mortality in Asia was 66.9% and 4.39 per 1,000 person-years, respectively, at the AMK, Bangladesh, site.

Table 1 shows mortality rates per 1,000 person-years for NCD causes. Age groupings follow the WHO 2012 categories (15–49, 50–64, 65+). NCD mortality rates were substantially lower in the 15–49 age group (range 0.31 to 2.76 per 1,000 person-years across all sites and periods) compared to the 50–64 age group (range 2.81 to 14.06 per 1,000 person-years) and more so compared to the 65-plus age group (range 10.72 to 97.20 per 1,000 person-years).

**Table 1:** NCD mortality rates per 1,000 person-years by site, age group and period

Age group	15–49 years			50–64 years			65+ years		

Period	<2000	2000–2005	2006–2012	<2000	2000–2005	2006–2012	<2000	2000–2005	2006–2012
Matlab, Bangladesh		0.81	0.83		6.33	7.41		37.55	41.27
Bandarban, Bangladesh			0.83			4.80			18.31
Chakaria, Bangladesh			0.77			7.09			35.58
AMK, Bangladesh		0.87	0.75		6.60	7.12		45.29	43.82
Nouna, Burkina Faso	0.70	0.48	0.27	7.11	3.64	2.80	23.22	16.81	9.67
Ouagadougou, Burkina Faso			0.63			5.87			21.29
Taabo, Côte d'Ivoire			0.67			3.77			16.91
Kilite Awlaelo, Ethiopia			0.49			1.52			12.32
Farafenni, The Gambia	1.07	0.85	0.65	7.61	6.93	4.06	24.57	29.12	21.58
Navrongo, Ghana		1.87	1.72		10.16	8.96		26.82	24.51
Dodowa, Ghana			0.84			5.55			19.07
Ballabgarh, India			0.56			6.00			30.44
Vadu, India			0.43			3.90			17.46
Kilifi, Kenya			0.65			5.45			27.68
Kisumu, Kenya		2.44	1.65		7.96	7.36		25.95	32.05
Nairobi, Kenya		0.45	0.61		4.21	2.76		12.64	19.37
Karonga, Malawi		1.63	0.79		5.70	4.70		27.81	23.31
Niakhar, Senegal		0.76	0.56		3.15	2.54		24.22	16.15
Agincourt, South Africa	0.53	1.02	1.25	4.01	6.88	7.79	17.10	22.48	27.26
Africa Centre, South Africa		1.26	1.06		10.54	10.50		36.86	36.93
FilaBavi, Vietnam			0.96			3.76			28.63

**Fig. 2:**
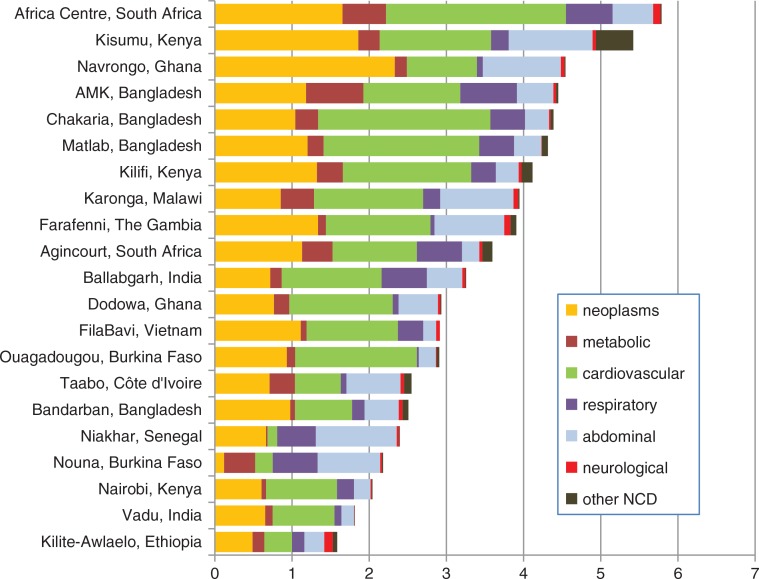
Age–sex–time standardised mortality rates per 1,000 person-years among adults (15 years and over) in 21 INDEPTH HDSS sites in Africa and Asia, by sub-category of non-communicable diseases causing death (according to WHO 2012 VA cause of death chapters).

Figure 2 shows the age–sex–time standardised mortality rates by site for sub-categories of NCD causes of death (neoplasms, metabolic, cardiovascular, respiratory, abdominal, neurological and other NCDs, according to chapters in WHO 2012 VA standard (34)) for 21 INDEPTH HDSS sites. These categories differ slightly from the four main NCD groups that are commonly considered in the global NCD literature, namely cancer, diabetes, cardiovascular disease, and chronic respiratory disease. Cancer and cardiovascular disease map directly to the WHO 2012 VA neoplasms and cardiovascular disease chapters, respectively. Diabetes accounted for 72% of deaths in the metabolic disease chapter, and chronic obstructive pulmonary disease accounted for 65% of deaths in the chronic respiratory disease chapter.

**Fig. 3:**
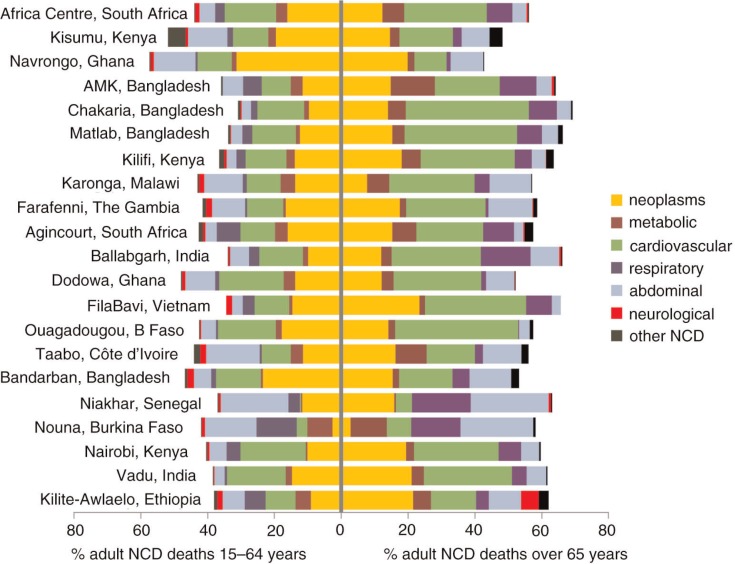
Age–sex–time standardised percentages of adult NCD deaths for the 15–64 and over 65 year age groups by site and cause category.

The population burden of premature NCD mortality has to be considered in terms of relative numbers of deaths between age groups, rather than only cause-specific rates, because of the very different population proportions in various age groups. While the mortality rate ratios for NCD mortality between the 15–64 and over-65 year age groups varied from 1:7 (Navrongo, Ghana) to 1:107 (Nairobi, Kenya), with a median of 1:18 (Niakhar, Senegal), this does not mean that the overwhelming burden of NCD mortality in terms of numbers of deaths lay within the over-65 age group. Person-time for the over-65 age group ranged from 1.4% of that for the 15–64 age group (Nairobi, Kenya) to 13.8% (FilaBavi, Vietnam), with a median of 8.2% (Chakaria, Bangladesh). NCD mortality rates for the 15–64 year age group ranged from 0.12 per 1,000 person-years in the Matlab, Bangladesh, site to 0.77 per 1,000 person-years in the Africa Centre, South Africa, site. Similarly for the over-65 age group, the range was from 1.24 in FilaBavi, Vietnam, to 13.4 in Nairobi, Kenya. Consequently, the numbers of NCD deaths in the 15–64 age group, which might be considered as ‘premature’ NCD mortality, were considerable. Figure 3 presents the percentages of adult NCD deaths for the 15–64 and over 65-year age groups by site, using the same cause categories as Fig. 2, to illustrate the magnitude of the burden of premature NCD mortality. The overall bar for each site represents 100% of NCD deaths, split by cause categories and age group. Consequently, bars relating to higher life expectancy countries, with higher proportions of older people, tend to be shifted further to the right. Slightly more than half of the overall adult NCD deaths occurred in the over-65 age group, to the right; deaths shown to the left of the axis might be considered as ‘premature’. Sites are shown in decreasing order of overall adult NCD mortality rate, the same as in Fig. 2, for ease of comparison.

Table 2 shows cause-specific rates at each site for the various categories of neoplasms specified in the WHO 2012 VA standard, separately for the under- and over-65 year age groups, and by sex, for the period 2006–2012.

**Table 2:** Cause-specific adult mortality rates for neoplasms (according to WHO 2012 VA cause categories), by site, age group, and sex for 2006–2012

	Oral neoplasms				Digestive neoplasms				Respiratory neoplasms				Breast neoplasms		Reproductive neoplasms				Other neoplasms			
	
Sex	Males		Females		Males		Females		Males		Females		Females		Males		Females		Males		Females	
Age (years)	<65	65+	<65	65+	<65	65+	<65	65+	<65	65+	<65	65+	<65	65+	<65	65+	<65	65+	<65	65+	<65	65+
Bangladesh: Matlab	0.01		0.01	0.04	0.45	3.84	0.23	2.35	0.38	5.41	0.12	2.80	0.03	0.06	0.03	0.56	0.05	1.34	0.19	1.95	0.05	1.89
Bangladesh: Bandarban			0.02		0.61	0.70	0.19	1.30	0.17	3.88		1.97	0.06		0.04		0.14	1.40	0.24	0.71	0.05	0.77
Bangladesh: Chakaria				0.2	0.16	1.82	0.08	1.38	0.16	4.47	0.15	2.36	0.02	0.37	0.00	0.69	0.02	1.01	0.30	2.55	0.18	1.72
Bangladesh: AMK		0.19	0.01		0.30	2.23	0.25	3.12	0.19	5.40	0.09	1.76	0.02		0.01	0.42	0.04	1.02	0.11	1.46	0.04	1.55
Burkina Faso: Nouna					0.03	0.35	0.01	0.11	0.02								0.00	0.23				
Burkina Faso: Ouagadougou	0.01		0.01		0.20	3.85	0.15	0.64	0.19	2.55	0.11	1.69	0.04		0.05	0.76	0.07	0.52	0.08	0.72	0.01	0.31
Cote d'Ivoire: Taabo	0.07	1.55			0.23	1.52	0.05	1.69	0.01	0.37	0.03		0.03	0.14		1.14	0.06		0.08	2.21		1.25
Ethiopia: Kilite Awlaelo					0.04	0.80	0.07	1.27	0.08	1.88	0.05	1.27				0.41	0.03	0.44	0.05	0.87		1.43
The Gambia: Farafenni		0.52			0.32	2.59	0.15	1.52	0.03	1.40	0.03	1.03	0.08		0.02	0.99	0.06		0.14	2.51	0.06	1.84
Ghana: Navrongo	0.04	0.38	0.03	0.09	1.57	9.51	0.89	5.25	0.29	3.78	0.11	1.38	0.14	0.34	0.13	1.08	0.15	1.80	0.05	0.33	0.01	0.29
Ghana: Dodowa		0.03	0.01		0.32	3.37	0.25	1.99	0.02	0.56	0.04	0.53	0.05	0.16	0.02	0.2	0.05	0.48	0.03	1.18	0.05	0.84
India: Ballabgarh	0.01	0.08	0.01	0.26	0.27	2.82	0.19	2.13	0.05	1.16	0.03	0.80		0.12	0.06	2.06	0.03	1.13	0.05	0.31	0.04	0.30
India: Vadu	0.01	0.47			0.27	5.95	0.10	1.17	0.04	1.80	0.05	1.08	0.06		0.02	0.07	0.07	1.43	0.02			0.19
Kenya: Kilifi		0.45	0.01		0.17	2.85	0.12	0.94	0.29	4.02	0.14	2.36	0.01		0.01	1.02	0.05	0.55	0.14	3.16	0.07	1.27
Kenya: Kisumu	0.03	0.06	0.02	0.10	0.39	3.33	0.31	2.01	0.36	5.11	0.27	3.19	0.04	0.07	0.01	0.34	0.08	0.55	0.46	3.47	0.19	3.31
Kenya: Nairobi		0.14	0.01		0.07	0.86	0.02	1.48	0.07	4.41	0.06	2.80	0.01	0.45	0.01		0.04	1.71	0.06	0.84	0.01	0.44
Malawi: Karonga			0.01		0.22	2.56	0.20	1.12		0.25	0.01	0.08	0.03	0.21	0.02	0.55	0.17	1.27	0.02		0.01	
Senegal: Niakhar					0.09	0.55	0.07	0.19		0.14	0.06	0.13	0.01			0.24	0.01	0.82	0.25	4.59	0.02	2.02
South Africa: Agincourt	0.01		0.01	0.09	0.24	1.29	0.17	2.69	0.26	2.70	0.19	2.68	0.08	0.13	0.02	0.24	0.15	0.68	0.12	0.99	0.07	1.69
South Africa: Africa Centre	0.03	0.05	0.02	0.07	0.38	1.88	0.18	0.86	0.34	5.42	0.20	1.62	0.06	0.27	0.04	1.31	0.23	1.47	0.18	2.31	0.07	1.03
Vietnam: FilaBavi			0.01		0.24	2.85	0.17	1.37	0.38	8.61	0.06	2.74	0.01		0.03	0.50	0.04	1.06	0.20	3.50	0.08	1.60

Table 3 shows cause-specific rates at each site for other selected categories of NCDs specified in the WHO 2012 VA standard, separately for the under- and over-65 year age groups, and by sex, for the period 2006–2012.

**Table 3:** Cause-specific adult mortality rates for selected NCDs (according to WHO 2012 VA cause categories), by site, age group, and sex for 2006–2012

	Diabetes mellitus				Acute cardiac disease				Stroke				Other cardiac disease				Chronic obstructive pulmonary disease			
	
Sex	Males		Females		Males		Females		Males		Females		Males		Females		Males		Females	
Age (years)	<65	65+	<65	65+	<65	65+	<65	65+	<65	65+	<65	65+	<65	65+	<65	65+	<65	65+	<65	65+
Bangladesh: Matlab	0.04	0.91	0.03	1.71	0.30	2.07	0.05	0.47	0.60	14.14	0.39	17.03	0.27	4.74	0.10	3.44	0.13	2.92	0.03	1.71
Bangladesh: Bandarban					0.35	3.75		0.33	0.15	3.15	0.16	2.69	0.06	0.35	0.13	0.73		0.71	0.04	2.64
Bangladesh: Chakaria	0.10	5.19	0.19	7.13	0.06	2.98	0.03	1.39	0.27	5.12	0.24	6.00	0.10	3.28	0.10	2.93	0.11	5.04	0.21	4.95
Bangladesh: AMK	0.09	2.61	0.08	2.26	0.40	2.31	0.06	1.55	0.43	14.38	0.31	16.82	0.35	6.98	0.22	5.06	0.09	5.63	0.06	2.65
Burkina Faso: Nouna	0.07	1.76	0.12	2.22		0.10	0.01		0.04	2.26	0.03	0.27		0.08		0.41	0.25	2.06	0.09	1.35
Burkina Faso: Ouagadougou	0.03	0.26	0.03		0.15	2.86	0.05	1.25	0.29	11.66	0.21	6.54	0.06	5.89	0.06	0.22				
Cote d'Ivoire: Taabo		0.51	0.07	1.12	0.02		0.03		0.10	2.87	0.09		0.07	5.63	0.11	0.53	0.03	1.49		
Ethiopia: Kilite Awlaelo	0.03	0.53	0.03	0.40	0.03	0.49	0.01	0.18	0.06	1.81	0.08	1.18	0.07	1.16	0.06	0.57	0.09	0.41	0.02	0.61
The Gambia: Farafenni		0.27	0.03		0.14	0.34	0.02	0.43	0.10	6.36	0.31	7.12	0.06	1.94	0.05	0.45	0.02	0.92		
Ghana: Navrongo	0.08	1.15	0.07	0.66	0.37	2.64	0.09	0.93	0.26	2.60	0.15	1.88	0.16	2.05	0.14	1.41	0.03	0.76	0.02	0.54
Ghana: Dodowa	0.04	0.78	0.01	0.39	0.24	4.30	0.17	2.60	0.27	4.93	0.30	5.09	0.06	1.48	0.09	1.34	0.01	0.10		0.08
India: Ballabgarh	0.05	1.67	0.05	0.81	0.22	4.38	0.20	1.63	0.26	7.78	0.13	5.38	0.12	3.96	0.07	2.12	0.09	8.54	0.09	4.39
India: Vadu	0.01	0.24	0.02		0.08	1.64	0.08	1.02	0.14	4.12	0.18	5.43	0.07	1.41	0.15	1.17	0.01	1.19		0.74
Kenya: Kilifi	0.04	1.91	0.02	0.73	0.03	0.92	0.02	0.29	0.29	7.00	0.24	7.16	0.15	5.94	0.14	3.84	0.05	1.92	0.03	1.25
Kenya: Kisumu	0.05	2.04	0.02	0.56	0.05	0.85	0.03	0.49	0.13	1.27	0.10	2.18	0.39	8.84	0.38	7.97	0.07	1.89	0.03	1.64
Kenya: Nairobi	0.01		0.00	0.44	0.10	1.16	0.04	1.58	0.04	0.50	0.03	1.81	0.27	5.61	0.27	6.25	0.03	1.23	0.00	1.39
Malawi: Karonga	0.02	1.97	0.08	0.58	0.08	1.06		0.83	0.22	6.41	0.23	7.12	0.04	2.80	0.04	2.76		1.47	0.01	1.72
Senegal: Niakhar						0.60		0.38		1.13		0.59		0.14	0.02	0.20	0.09	5.15	0.03	4.03
South Africa: Agincourt	0.14	1.83	0.12	6.57	0.07	0.29	0.04	0.60	0.25	2.53	0.24	11.42	0.17	2.15	0.19	8.77	0.22	1.69	0.11	4.71
South Africa: Africa Centre	0.18	4.22	0.25	4.20	0.12	0.75	0.06	0.34	0.23	6.36	0.26	6.58	0.33	8.82	0.47	9.79	0.06	4.14	0.08	4.09
Vietnam: FilaBavi	0.02	0.36		0.28	0.06	1.40	0.02	0.42	0.23	8.27	0.12	10.04	0.32	5.00	0.14	2.83	0.02	1.11		0.69

**Fig. 4:**
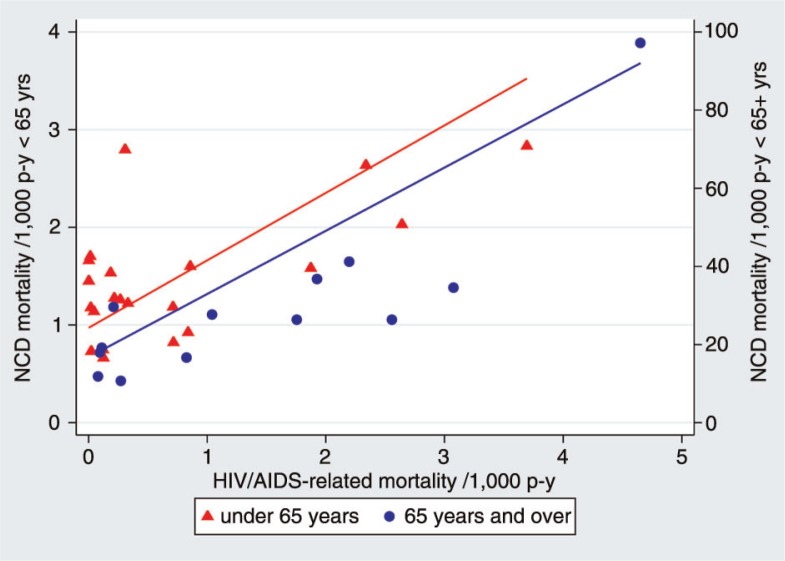
Age–sex–time standardised NCD mortality for 15–64 and over-65 year age groups in relation to age–sex–time standardised HIV/AIDS-related mortality in the same populations, all per 1,000 person-years.

Because some of the sites reporting the highest rates of NCD mortality were also those with the greatest burden of HIV/AIDS-related mortality (as explored from the same dataset in a separate paper (35)), in Fig. 4 we present, separately for the 15–64 and over-65 year age groups, the correlations between NCD and HIV/AIDS mortality rates, showing points for each site and least absolute deviations regression lines for both age groups. In both age categories, correlations are significant (p<0.005; R^2^=0.40 and 0.37, respectively) and suggest that in high HIV settings, around half of the mortality attributed to NCDs may well be associated with HIV. This was also reflected in falling NCD mortality rates among younger adults at most African sites between the 2000–05 and 2006–12 periods (Table 1), as HIV/AIDS-related mortality reduced sharply (35).

## Discussion

This study reports NCD mortality patterns for a large number of adults across a wide range of settings in Africa and Asia. Being part of the overall INDEPTH cause-specific mortality study (8), these rates for NCDs constitute a component of complete mortality surveillance in each site, as distinct from studies only considering NCDs. The age–sex–time standardised results presented here enable comparisons to be made between sites allowing for the dynamics of age–sex population profiles in different places.

Although proportions of NCD mortality were generally higher in Asian sites (Fig. 1), rates of NCD mortality were more consistent across African and Asian sites. This observation is driven less by NCD-specific factors than by demographic transition. Many populations in Asia are experiencing marked increases in mid-life survival, and consequently life expectancy, which lead to historically low levels of crude mortality. Thus, according to UN Population estimates, many Asian countries currently record much lower crude mortality rates than their European and American counterparts, having reduced infection-related mortality but not having yet accumulated substantial population proportions of older people (36). As populations gain larger numbers of older people, there will be increasing numbers of NCD-related deaths, even if rates remain the same.

Because not all sites operated population surveillance for the same time period, we have limited evidence on the time trends in NCD mortality rates (Table 1). However, in the sites where trends could be seen, there was little clear evidence of NCD mortality rates increasing over time. As expected for NCD mortality, rates increased very markedly with age; however, the relatively small population proportions of people over 65 years in most of these sites means that the high mortality rates observed in that age group do not yet translate into large numbers of deaths.

Figure 2 shows that cancers and cardiovascular diseases were the major components of NCD mortality in almost all sites. Similar proportions of most causes were seen across most sites; a notable exception was the lack of cancer-related mortality at the Nouna, Burkina Faso, site, which is possibly a consequence of items missing from the VA instrument in use there. Some cancer deaths at Nouna were probably misclassified into the relatively high rates of respiratory and abdominal NCDs at that site. This is an example of some of the difficulties that may occur as a result of harmonising VA data collected at many sites, over different time periods, using a variety of antecedents to the WHO 2012 standard (37). This is discussed further in the introductory paper (8) but there are no simple solutions.

Table 2 shows a breakdown of cancer-related mortality rates. As one of few long-established national cancer registries in sub-Saharan Africa, the Gambian cancer registry has reported incidence rates for various cancers over more than 20 years, partly with the aim of tracking possible reductions in liver cancer incidence following the pilot introduction of hepatitis B vaccination in the 1980s (38). Although incidence and mortality rates are not directly comparable, there are similar age–sex patterns in the Gambian incidence rates and the mortality rates reported here, particularly for several of the West African sites. In eastern and southern Africa, cancer mortality rates may also be influenced by high HIV rates (39). Respiratory cancer mortality as reported here showed higher rates for men in most sites over both age groups, which probably reflects gendered norms around smoking as a risk factor. Appreciable rates of ‘other neoplasms’ were recorded from many sites, which may partly reflect a lack of specific information about cancers recalled in VA interviews, or a lack of specific items in some historic VA instruments.

Table 3 shows, in the same format as Table 2, mortality rates for diabetes, cardiovascular disease, and chronic obstructive pulmonary disease. These categories were major contributors to NCD mortality across many sites, although the relative magnitude of these categories of mortality varied between sites. Some sites were using VA instruments designed before NCDs were considered to be major causes of death in the populations concerned. It is likely that the quality of NCD cause assignment achievable by InterVA-4 will increase as primary VA data collected according to the complete WHO 2012 VA specification become more widely available. Nevertheless, the overall consistency observed across these varied sites in many respects suggests that historical inter-site variations did not have a major influence on the overall pattern of NCD mortality findings.

The importance of separating NCD mortality into premature and less-avoidable groupings by age is clearly illustrated in Fig. 3. Due consideration must be given to both NCD mortality rates as well as numbers of NCD deaths in considering how disease burdens may be changing. Here, we have somewhat arbitrarily taken 65 years as the cut-off for considering premature mortality, because that fitted with the WHO 2012 VA age groups used in this dataset. In addition, many of the countries represented here have life expectancies at birth in the range of 60 to 70 years, and so 65 years may represent a reasonable dichotomy in terms of ‘prematurity’. In the global context, 70 years might be a more reasonable cut-off (40). As yet, none of the countries represented here has a top-heavy population pyramid, with the consequence that there is a reasonably even balance in proportions of NCD deaths occurring before and after 65 years of age, despite high NCD rates among older people. However, it is to be expected, as time passes, that there will be a gradual rightward shift in the pattern shown in Fig. 3, unless increased NCD risk factor exposures at younger ages also increase NCD mortality rates among the under-65 age group. Nevertheless, the appreciable number of NCD deaths recorded among the under-65 age group must also reflect a considerable burden of chronic disease morbidity in that otherwise productive age group. To the contrary, modelling results suggest that it could be possible to achieve significant global reductions in premature (under-70 years) NCD deaths if relevant risk factors were appropriately managed (40); however, the extent to which those risk factors are likely to be effectively managed in the countries reported here remains an important question.

The contribution of diabetes to the overall picture of NCD mortality in these populations was relatively small compared to cancers and cardiovascular diseases. Whether this reflects situations where risk factors are not yet translating into mortality rates, or whether VA methods led to some misclassification of diabetes mortality into, for example, cardiovascular deaths is uncertain.

The correlations between HIV-related mortality and NCD mortality across the whole adult age range, as shown in Fig. 4, show how important it is to take a holistic view of cause-specific mortality across entire populations. The increased NCD mortality rates in higher HIV mortality populations here are consistent with previous work that analysed causes of death by HIV status (39). This is clearly an important factor to bear in mind when comparing patterns of NCD mortality across sites with varying HIV epidemiology. The ICD-10 system tries to classify almost all deaths in HIV-positive people under the B2 categories related to HIV (41). In reality, however, particularly if HIV status is not known and for final illnesses that are not indicative of HIV/AIDS, it is very likely (by any means of cause of death assignment) that a proportion of deaths among HIV-positive people will be attributed to NCDs. This is likely to be an increasing issue as higher proportions of HIV-positive people receive effective antiretroviral treatment.

## Conclusions

Against a background of considerable concern about the magnitude of the global NCD mortality burden, these population-based findings provide useful geographic examples of the state of adult NCD mortality across a range of locations in Africa and Asia, standardised for ease of comparison. The importance of distinguishing between premature NCD mortality and the inevitably high rates of NCDs that occur late in life is clear from these data. These findings also provide a useful baseline from which to track future trends in NCD mortality, using the same standardised methods. It is also evident that, in areas of high HIV endemicity, NCD mortality rates have to be seen as possibly including some HIV-related deaths, in cases where no clear evidence of HIV status was available.
